# Copper Amalgam

**Published:** 1892-07

**Authors:** S. B. Palmer

**Affiliations:** Syracuse, N. Y.


### COPPER AMALGAM.[1]

BY DR. S. B. PALMER, SYRACUSE, N. Y.

You ask for my conclusions regarding copper amalgam. I am glad to exchange my views, which are from a scientific stand-point, with others of more practical knowledge. The following reasons have hindered the use of copper amalgam in my practice to *an extent* that I very much desire to be set right, if in error, and thus secure the benefits which this material offers.

I cannot express my conclusions without giving some of the principles upon which they are based. Copper amalgam differs from that ordinarily used, in this particular: it is composed of two elements, copper and mercury, while that generally used is composed of an alloy containing two or more metals, which is of importance, as may be seen farther on. Copper amalgam must be unreliable that is not uniform in its tooth-saving properties, for the following reasons: Two metals are united in a mass and exposed to fluids which are liable to dissolve either copper or mercury, or to act upon the two, and by such action polarize the elements or form a protecting compound surface, which the fluids will not dissolve. In this case only is copper amalgam a success.

In the process of amalgamation neither metal loses its identity. When the fluids find affinity in copper it is dissolved,—the surface of such plugs remains white, the fillings are soft and waste rapidly. When mercury is taken away the surface of the plug looks brown and spongy. With amalgam made from alloy there is this advantage: instead of the electro-chemical change being confined to one ele-

---

[1] Read at a meeting of the New York Odontological Society, held April 19, 1892.

ment, it is shared by all that compose the plug, including the mercury. To explain: in copper amalgam there are only two metals; either is liable to the positive one in an alloy, say, of silver and tin; the mercury enters into the compound to make comparatively one of three instead of two elements,—that is, there is little or no free mercury. Let us imagine there is mercury which is not absorbed or satisfied by the other metals, the same holds different relations to the plug proper from that of pure copper, because by the amalgamation of the silver and tin, an element is present containing mercury which greatly reduces the potential.

In good amalgams the plugs are not dissolved beneath a surface of mercury.

Copper amalgam may be heated and the two metals so separated as to become plastic; not so with alloys, because mercury is a part of the whole. The operations of a simple battery teach a lesson well worth notice. The object of a battery is to furnish a current. The requirements of an amalgam plug is to avoid a current, or dissolution of the elements. Commercial zinc contains other metals which, by local currents, oxidizes the surface and retards action. The surface of the plate is amalgamated, and in effect there are two elements,—mercury and zinc,—whereas in copper amalgam it is mercury and copper; add to the zinc silver, tin, copper, etc., the mercury would be taken up and the element become useless for battery purposes.

The above reasons have prevented the introduction of copper amalgam in my practice.

